# Supplementary material for: The Drosophila orthologue of the primary ciliary dyskinesia-associated gene, DNAAF3, is required for axonemal dynein assembly
Source: Biol Open. 2021 Oct 28;10(10):bio058812. doi: 10.1242/bio.058812 (PMC8565470; doi:10.1242/bio.058812)
Supplement: Supplementary information [file biolopen-10-058812-s1.pdf]

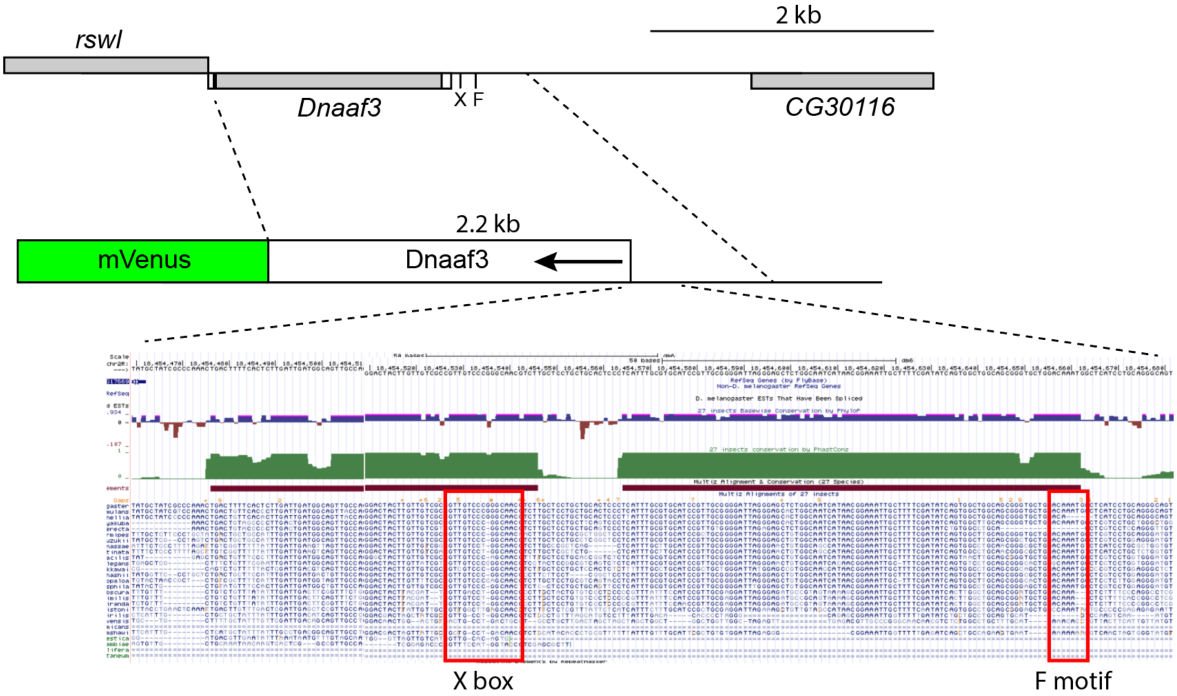

Fig. S1. *Dnaaf3-mVenus* fusion gene was designed to include the X box (consensus nucleotide sequence RYYRYYN(1–3)RRNRAC) (Laurençon et al., 2007) and Forkhead binding motif (F motif) (consensus nucleotide sequence RYMAAYA) (Newton et al., 2012) motifs in the upstream regulatory region to keep the spatial-temporal pattern of the expression from its own promoter.

Biology Open • Supplementary information

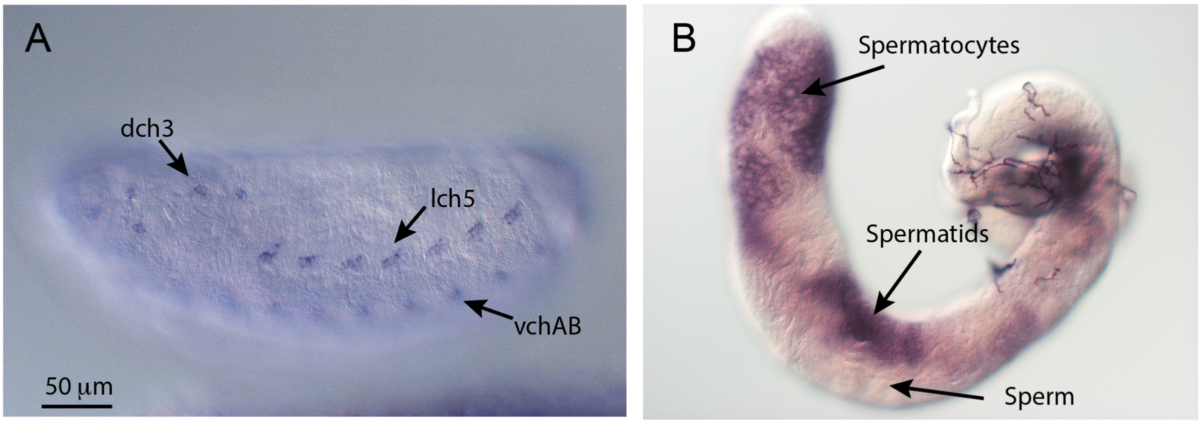

Fig. S2. *Dnaaf3* mRNA expression. (A) Stage 16 embryo. Expression is observed in the segmentally repeated locations expected for larval Ch neurons (see Fig. 1B). (B) Adult testis. The large nuclei identify the expressing cells near the tip as spermatocytes. Expression further along is likely to be in differentiating spermatids. No expression is seen in mature sperm.

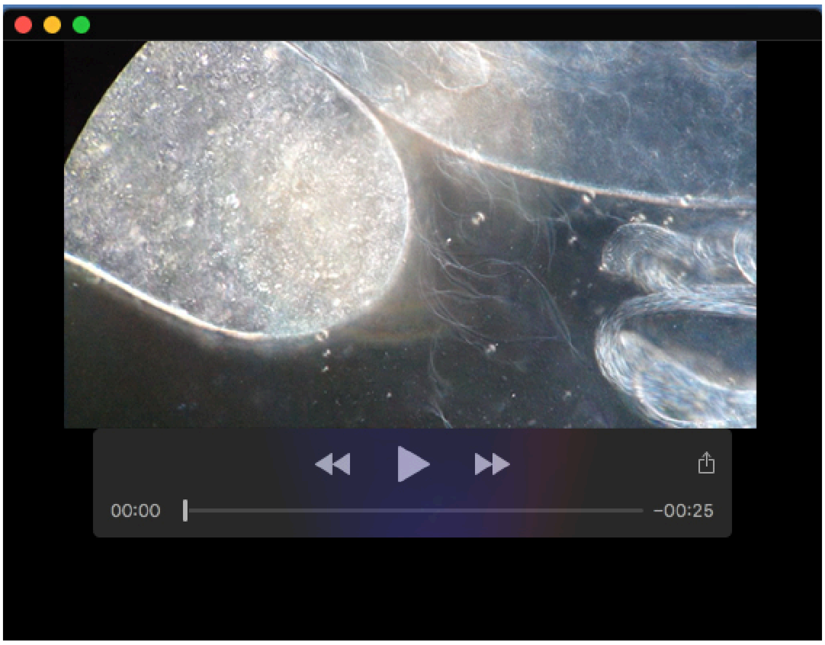

Movie 1. Sperm from *Bam*Gal4 fly (control for RNAi)

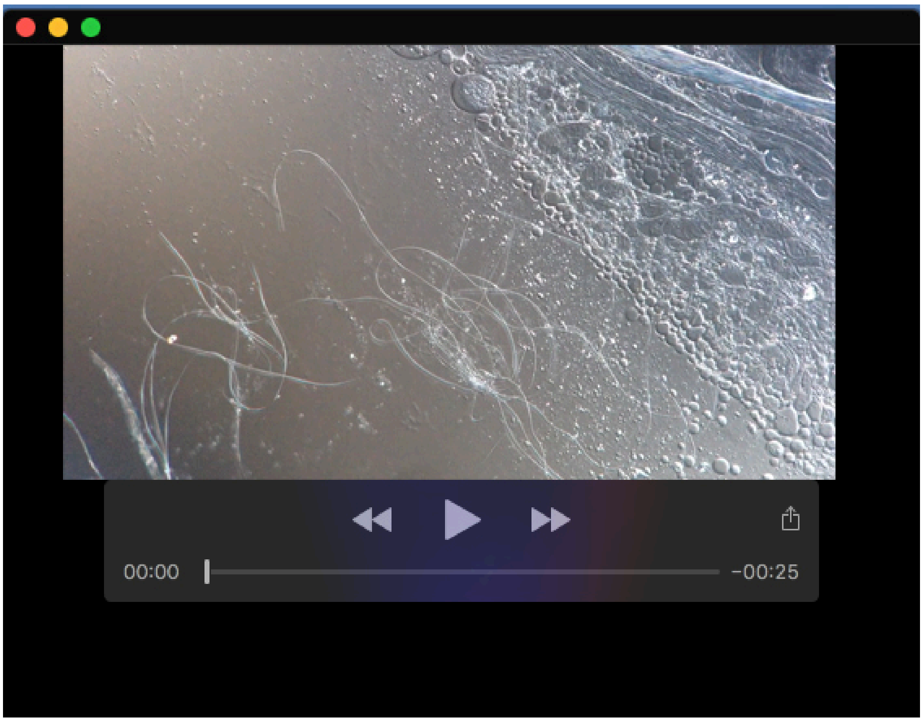

Movie 2. Sperm from *Bam*Gal4; UAS-*Dnaaf3* RNAi fly (*Dnaaf3* knockdown)

Biology Open • Supplementary information

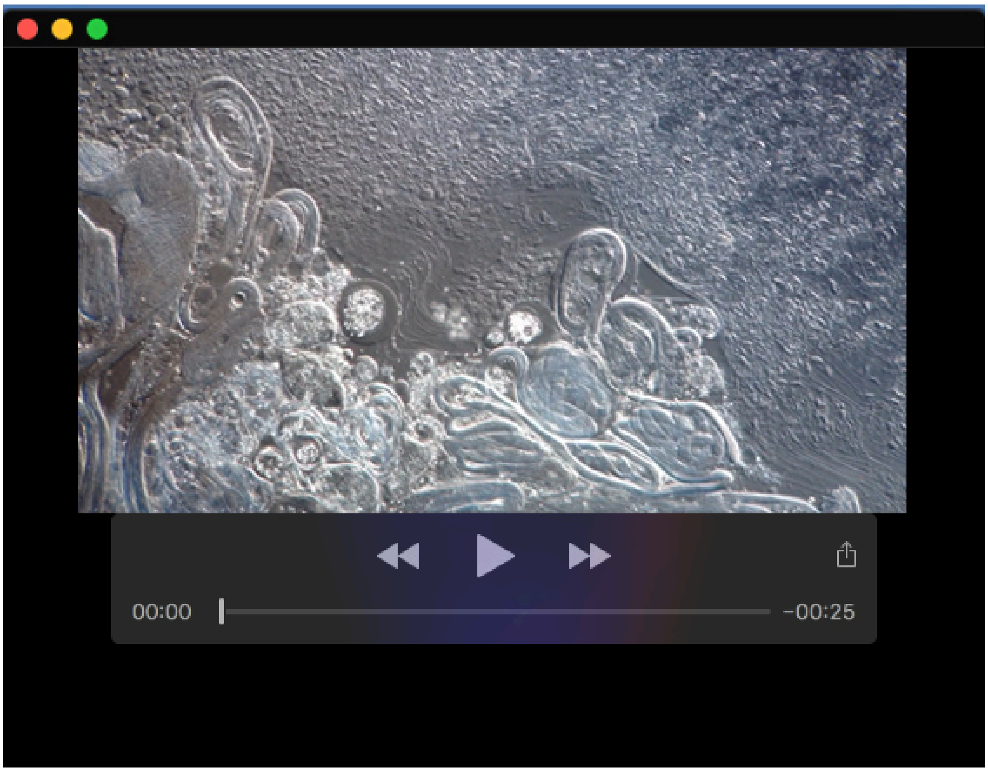

Movie 3. Sperm from *Dnaaf3*$^{\Delta CR}$ homozygote

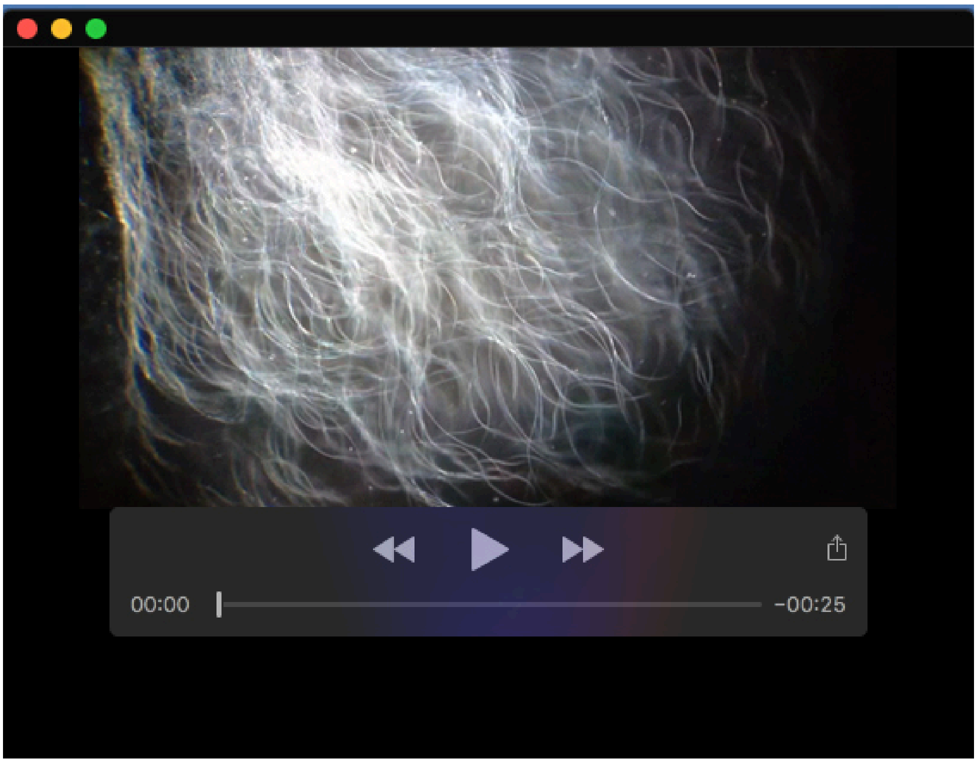

Movie 4. Sperm from *Dnaaf3*$^{\Delta CR}$ homozygote with *Dnaaf3-mVenus* fusion gene (mutant rescue)

Biology Open • Supplementary information

Table S1. Ciliary motility proteins detected in adult testes, sorted by abundance

Majority protein IDs	Gene names	Average LFQ control*	Average LFQ CG17669*	-log2 ratio	-log10 p value
Q9VH07	pont	9.87783848	9.62517004	0.037383	0.032098
Q8INT5	Dnaaf1	9.14370354	11.50210626	-0.33105	0.265354
Q5LJN5	kl-5	8.19193092	2.814315855	1.541419	2.208549
Q9V3K3	rept	7.88284253	8.65178034	-0.13428	0.235542
Q9V3M9	Tektin-A	7.25402673	2.979805372	1.283564	1.186591
A8Y5B7	kl-3	6.80093659	0.430428367	3.981888	3.182219
Q9VBA1	Spag1	6.39372796	6.470997003	-0.01733	0.011752
Q9VU41	Zmynd10	5.59104156	7.163702465	-0.35759	0.363388
Q9W1V2	CG3085	4.91455102	1.939847136	1.341117	1.576174
Q9VGG6	Dnali1	3.88414271	1.440937117	1.430589	1.638454
Q9VGC1	CG10014	3.6990868	2.098445308	0.817848	0.688281
Q9V3E9	Rpap3	3.05702371	3.072928769	-0.00749	0.0058
Q9W1D3	Rsph4a	3.04296406	2.084000363	0.546122	0.926048
Q9VJC6	Dhc36C	2.82836711	0.163285342	4.114502	2.494444
Q8T3Z0	Tektin-C	2.44201942	1.224471976	0.995915	1.457508
Q8INF7	Heatr2	2.35414183	2.489974686	-0.08093	0.169468
Q9VT21	CG8336	2.33143917	2.505177094	-0.10369	0.116157
Q9VAV5	Dhc98D	1.83012501	0.712779034	1.360415	2.116203
Q9VM21	gudu	1.62031671	0.232234203	2.802623	2.02667
Q9VUG3	Pih1D3	1.57346805	1.741292636	-0.14621	0.1014
Q9VZH1	CG18675	1.47753788	1.76610829	-0.25738	0.309647
Q9VN57	CG17387	1.41627716	1.324956294	0.096159	0.070752
Q24117	ctp	1.36907896	1.078564964	0.344093	0.207812
Q9VK29	Rsph1	1.35813245	0.991262186	0.454286	0.621901
**A1ZB91**	**CG17669/Dnaaf3**	**1.2915202**	**0.081774953**	**3.981267**	**3.103016**
Q0E8T7	CG34124	1.18667573	0.490089781	1.275808	2.25354
Q8T415	Centrin	1.16685798	0.984236486	0.245552	0.207075
Q9VYR5	CG18130	1.10884709	0.581556947	0.931068	0.909879
Q9VZ77	Dnah3	1.08739055	0.119223228	3.189133	2.309416
Q9VCN4	Ccdc114	1.08124333	0.208368775	2.37548	1.746445

Biology Open • Supplementary information

Q94524	Dlc90F	1.08013754	0.998358295	0.113585	0.088463
Q8MT08	Gas8	1.05840147	0.268884548	1.976828	1.168448
Q9VWZ3	Dhc16F	1.01829888	0.041609539	4.613103	3.265697
Q7KVA7	Dhc62B	1.00184398	0.157084244	2.673047	2.321502
Q9VRY7	CG10099	0.91960338	0.606549278	0.600387	0.664016
Q8T3V7	Rsph9	0.86288384	0.617610339	0.482469	0.659223
Q9VC34	CG6980	0.83779304	1.043301876	-0.31649	0.301679
Q5LJP0	kl-2	0.78943234	0.224258622	1.815652	2.822993
A0A0B7P7M6	l(2)41Ab	0.76685658	0.609264688	0.331888	0.30116
Q9VS90	Wdr63	0.76016435	0.510863279	0.573374	1.099499
Q9VJY4	Dnai2	0.67582694	0.303152945	1.156608	1.965192
Q9VA28	CG15547	0.55961706	0.304514523	0.877929	0.757275
Q9VU57	Cfap43	0.55106365	0.068451057	3.009074	2.674799
Q0E9G3	Dnaaf2	0.47060279	0.581374511	-0.30496	0.22944
Q4V516	lobo	0.4429829	0.227454095	0.961676	1.597102
Q9W3J8	CG10958	0.3214128	0.131678316	1.28741	1.370424
Q9VVM7	Wdr92	0.27889713	0.453183439	-0.70036	1.994956
Q9W0F0	robl62A	0.25048477	0.039268251	2.673288	1.774009
Q4QPW2	CG10822	0.23635937	0.124063924	0.929898	0.591768
Q9W0U9	Dic61B	0.22810593	0.14967333	0.607887	1.814405
A1Z8T9	CG8407	0.21119504	0.090270929	1.226243	0.634525
Q7KMS3	robl	0.17293244	0.22301487	-0.36693	0.193481
Q0KI05	Dnah9	0.15915179	0.020345612	2.967614	0.376532
Q9W2A3	CG13501	0.13118274	0.07513098	0.804098	1.408852
Q9W168	CG16837	0.06873574	0.028035895	1.293785	1.453004
Q9VKJ5	Dyx1c1	0.05987797	0.20546445	-1.77879	2.303495
Q9V9P1	CG10834	0.04958531	*5.00819E-10*		
Q7K4X4	Cfap53	0.04266028	0.11420404	-1.42065	0.929194
Q9VIY1	CG10750	0.03495738	0.007402311	2.23955	1.554517
Q9VK58	Pih1D1	0.03250981	0.03428731	-0.0768	0.097999
Q9W3L0	Dnai2	0.02907692	0.004135815	2.813631	0.631246
Q9VQA6	robl22E	0.02688946	0.036787338	-0.45217	0.136057
Q9VR52	Lrrc6	0.02411973	0.09266751	-1.94185	1.793124

Biology Open • Supplementary information

Q7KUM9	CG7276	0.01382293	0.005903806	1.227346	0.44837
A1Z8V5	Iqcd	0.01227507	0.001742399	2.816585	1.135211
A0A0B4LH20	CG9492	0.00647783	0.006053899	0.097646	0.016186
Q4V5H1	CG17266	0.0048374	0.007403708	-0.61402	0.278398
Q9VJ18	robl37BC	0.00099315	*5.00819E-10*		
P47947	TpnC41C	*5.7109E-10*	*5.00819E-10*		
A1Z8J9	CG13202	*5.7109E-10*	0.017887853		
A8JUM9	Casc1	*5.7109E-10*	*5.00819E-10*		
Q9VJC5	Maats1	*5.7109E-10*	*5.00819E-10*		

* italics represent imputed values. Proteins in red were excluded from further analysis due to very low detection level (average LFQ in control <0.05).

Biology Open • Supplementary information
